# Cost-effectiveness of community diabetes screening: Application of Akaike information criterion in rural communities of Nigeria

**DOI:** 10.3389/fpubh.2022.932631

**Published:** 2022-07-25

**Authors:** Anayochukwu Edward Anyasodor, Ezekiel Uba Nwose, Phillip Taderera Bwititi, Ross Stuart Richards

**Affiliations:** ^1^School of Dentistry and Medical Sciences, Charles Sturt University, Orange, NSW, Australia; ^2^Department of Public and Community Health, Novena University, Kwale, Nigeria

**Keywords:** fasting blood glucose, diabetes, prediabetes, screening, Nigeria, Akaike information criterion

## Abstract

**Background:**

The prevalence of diabetes mellitus (DM) is increasing globally, and this requires several approaches to screening. There are reports of alternative indices for prediction of DM, besides fasting blood glucose (FBG) level. This study, investigated the ability of combination of biochemical and anthropometric parameters and orodental disease indicators (ODIs) to generate models for DM prediction, using Akaike information criterion (AIC) to substantiate health economics of diabetes screening.

**Methods:**

Four hundred and thirty-three subjects were enrolled in the study in Ndokwa communities, Delta State, Nigeria, and their glycaemic status was determined, using the CardioChek analyser^®^ and previous data from the Prediabetes and Cardiovascular Complications Study were also used. The cost of screening for diabetes (NGN 300 = $0.72) in a not-for-profit organization/hospital was used as basis to calculate the health economics of number of individuals with DM in 1,000 participants. Data on the subjects' anthropometric, biochemical and ODI parameters were used to generate different models, using R statistical software (version 4.0.0). The different models were evaluated for their AIC values. Lowest AIC was considered as best model. Microsoft Excel software (version 2020) was used in preliminary analysis.

**Result:**

The cost of identifying <2 new subjects with hyperglycemia, in 1,000 people was ≥NGN 300,000 ($ 716). A total of 4,125 models were generated. AIC modeling indicates FBG test as the best model (AIC = 4), and the least being combination of random blood sugar + waist circumference + hip circumference (AIC ≈ 34). Models containing ODI parameters had AIC values >34, hence considered as not recommendable.

**Conclusion:**

The cost of general screening for diabetes in rural communities may appear high and burdensome in terms of health economics. However, the use of prediction models involving AIC is of value in terms of cost-benefit and cost-effectiveness to the healthcare consumers, which favors health economics.

## Introduction

The number of diabetics has sharply increased globally over the last three decades and, this makes the disease a significant public health issue. The prevalence of DM seems dependent on the level of socioeconomic development, with reports showing the prevalence higher in low-to-mid income countries (LMICs) than in high-income countries. There have been significant epidemiological changes in occurrence of type 2 DM (T2DM) over the years, especially its relative rarity in Africa some decades ago (1) but it is now reported that 80% of T2DM reside in developing communities (2). The prevalence of DM in Nigerian rural areas is 0–2%, and 5–10% in the urban (3–6). This study is mindful that the differences in prevalence may be attributed to different diets and lifestyles as well as availability of healthcare facilities, e.g., for screening and management in rural and urban communities. Regardless, this calls for early and affordable screening to mitigate complications associated with unmanaged or poorly controlled DM such as cardiovascular diseases.

In developing countries, the cost-effectiveness of screening for T2DM remains unknown (7), and health economic evaluations that compare screening alternatives are needed (8). Diabetes places additional burdens on patients and families of affected individuals (9), leading to enormous socioeconomic loss (10). Screening for DM is recommended to reduce the burden of the disease (11) and early detection and treatment seems a logical preventive approach for cost-saving among other reasons (7). While it is reported that diabetes screening is cost-effective (12), several factors define cost-effectiveness, hence, it is suggested that screening high-risk individuals is worthwhile (13). This study aims to inform decision makers about the potential health economic value of non-invasive methods in community diabetes screening.

Health information technology has the potential to improve the performance of service delivery, increase health care quality, save costs and engage patients as effective partners of their health care. There are opinions on application of information criterion to obtain optimal choice between models and among the options, Akaike information criterion (AIC) has received special attention (14). AIC is a measure of relative quality of statistical models for a dataset, and, is applied to ascertain the best model fit. A series of models are compared for AIC to become useful, and the model with the lowest AIC is considered the best. Estimation of effect of AIC, and its precision are important aspects of modeling, and not the declaration of significance. The AIC offers relative estimate of the information lost, when a given model is used to represent the process that generates the data. It takes into account the trade-off between goodness of fit of a statistical model and the complexity of the model (15).

Orodental disease indicators (ODIs) signal periodontitis and the later may increase the risk for aggravating glycaemic control in diabetic subjects. Periodontitis may also be implicated as risk for diabetic complications (16), and this suggests the need for consideration of ODIs as parameters to predict diabetes. However, what constitutes the best parameter to predict diabetes is contestable, as some reports indicate that anthropometric indices can predict diabetes (17–19). This led to the evaluation of AIC with other anthropometric parameters to ascertain the indices that predict diabetes better. Application of AIC in community-based screening in Nigeria is one way to improve diabetes control, especially in rural communities, hence the importance of this study.

## Methods

The study was carried out in Ndokwa communities of Nigeria, with the approval (protocol number: 2015/286) of Human Research Ethics Committee (HREC) of Charles Sturt University, Australia. The HREC of Novena University and Ndokwa West Local Ministry of Health Department also approved the research. Awareness and public lecture to the community about the study preceded the data collection. Information sheets were given to potential participants who gave consent to take part in the study. Data collection occurred at Catholic Hospital Abbi and Eku Baptist Government Hospital, both in Delta State of Nigeria and participants were Ndokwa residents of all sexes aged 18 years and above.

The World Health Organization (WHO) STEPS questionnaire elicited information from the subjects on presence or absence of pathological problems in their mouth/teeth. The ODI questions sought information on pain/discomfort, sleep interruption and feeling tensed due to state of the teeth/mouth. Other ODI questions involved description of the state of the gum/teeth and difficulties in biting/chewing food.

Biochemical measurements such as fasting blood glucose (FBG) and lipid profiles were carried out using CardioChek^®^ analyser, according to manufacturer's instructions. The criterion for diagnosing DM was ≥7.0 mmol/L (≥126 mg/dL) for FBG or >11.1 mmol/L (≥200 mg/dL) for random blood glucose and prediabetes was defined as impaired fasting glucose (IFG) level of 5.6 to <6.9 mmol/L (100 to <126 mg/dL) (20, 21). Measurands for lipid profile were blood high density lipoprotein cholesterol (HDL-C), total cholesterol (TC) and triglyceride (TG) concentrations. Cut-off values were 1.0 mmol/L (≤40 mg/dL) indicating low HDL in men and 1.3 mmol/L (≤50 mg/dL), indicating low HDL in women, 5.2 mmol/L (≥200 mg/dL) for hypercholesterolemia and 1.7 mmol/L (≥150 mg/dL) for hypertriglyceridemia as per classification of the International Diabetes Federation (IDF) (22).

The waist and hip circumferences were measured, using the ergonometric circumference measuring tape (Seca 203) and the procedures of IDF (22) were used to calculate the waist-hip ratio (WHR). Guidelines of WHO were followed to ensure accuracy of measurements and the recommendations for cut-offs for WC and WHR were observed (23). The cut-offs for this study were based on Europid values, and were: waist circumference >94 cm (men) and >80 cm (women) and for WHR, the value for men was ≥0.90 and ≥0.85 cm for women (22).

Statistical analysis followed three steps. First was modeling to identify a cost-effective model that identifies more DM. Various anthropometric, biochemical and ODI parameters were used to generate different models, using R statistical software (version 4.0.0). Only the prediabetes group was used in the modeling as per understanding that prediabetic state precedes frank DM. Data were collected from 433 subjects, and different models numbering 4,125 were generated.

Second step was determination of the best model and the top 400 out of 4,125 were selected and sorted, based on the AIC value. This was carried out to select the best models for prediction of prediabetes. Thirdly, to establish cost-effectiveness of community DM screening, the incidence was determined to identify the number of new individuals with DM and was particularly used to estimate whether it was cost-effective to spend Nigerian naira (NGN 300) to do universal screening in general population—i.e., average of how much would be leading to newly identified diabetics relative to the population of Ndokwa communities (149,325). The indicated amount above (NGN 300) is the cost of FBG test in a not-for-profit hospital, as observed during this study, but costlier in private health facilities.

Calculation of incidence was given by the formula:


$$\begin{array}{l} \text{Incidence rate}=\frac{\text{Number of new cases of disease in a period}}{\text{Sum of person}-\text{time at risk}} \end{array}$$

## Results

Table 1 is a simplified cost-effectiveness analysis of community-based diabetes screening. The incidence of prediabetes and UDM were 0.113 and 0.158%, respectively, or 0.159% hyperglycemia. The cost of identifying <2 persons with prediabetes or diabetes by screening a population of 1,000 was NGN 300,000. This serves as point of reference for not-for-profit hospitals and organizations, considering mass screening.

**Table 1:** Simplified cost-effectiveness analysis.

**Considerations**	**Glycaemic status**	
	**Prediabetes (%)**	**Diabetes (%)**
Previous prevalence of hyperglycemia		
Oguoma et al. (24)	4.9	5.4
Prevalence rate relative to 433 people screened	38.8	18.0
Incidence rate relative to Ndokwa population	0.113	0.046
Health economics cost of diabetes screening	NGN 300 ($ 0.72) in not-for-profit hospitals.	
Health economics cost of screening every 1,000	≥NGN 300,000 ($ 716) will be spent to identify <2 people persons with prediabetes or UDM	

*UDM, Undiagnosed diabetes mellitus, N = 433*.

**Figure 1:**
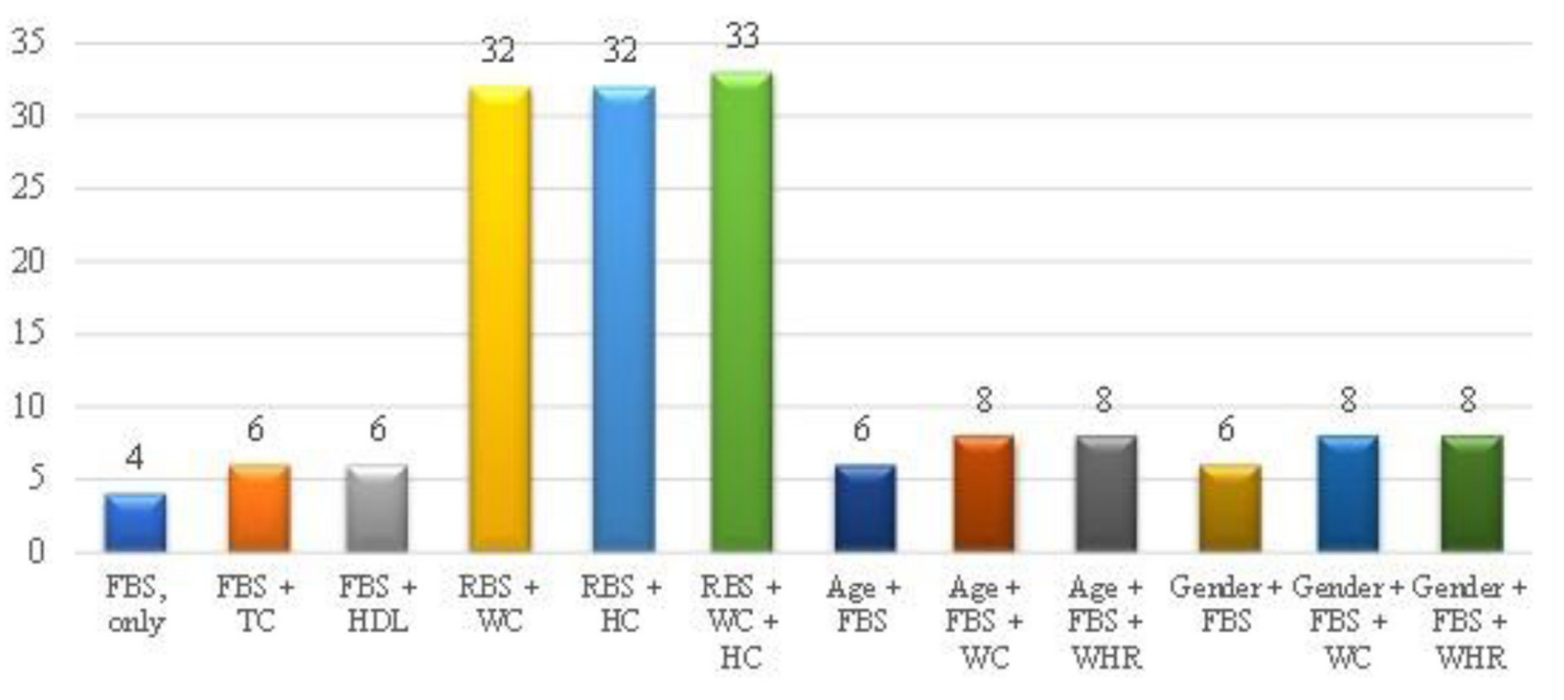
Modeling of anthropometric as well as biochemical variables and ODI scores of the overall study population. FBS (FBG), Fasting blood glucose; TC, Total cholesterol; HDL, High density lipoprotein; RBS, Random blood sugar; WC, Waist circumference; HC, Hip circumference; WHR, Waist-to-hip ratio. The y-axis of this figure represents the AIC value, while x-axis represents the parameters.

Figure 1 indicates modeling of anthropometric as well as biochemical variables and ODI scores in the prediction of diabetes. Based on AIC, the best model remained FBG due to it having the least AIC of 4, and the least was a combination of RBS + WC + HC (AIC = 33).

## Discussion

In a previous report from this research program, incidence of prediabetes and undiagnosed DM (UDM) was calculated at 38.8 and 18.0%, respectively. Oguoma et al. (24) determined the prevalence of prediabetes (4.9%) and diabetes (5.4%), and this formed the basis of the calculated incidence rate. The burden of diabetes including cost in Sub-Saharan Africa (SSA), including Nigeria is expected to worsen owing to rapid urbanization and aging population (25).This is evidenced by the incidence of hyperglycemia (prediabetes: 0.113 and diabetes: 0.046%) in this study (Table 1). The observation shows that regardless of diabetes intervention through education in Ndokwa communities, the incidence of the disease is rising. This therefore calls for community screening, thus first determination of a cost-effective approach to screening for UDM.

Anthropometric indices such as WHR, WC, age and gender as well lipid profile that includes blood TC and HDL levels are employed in predicting diabetes (26). A study in the USA showed WC to be a better predictor than WHR for DM (27). On the other hand, it has been suggested that WHR is an excellent predictor for the development of T2DM (28). The predictive abilities of WC, WHR, and BMI were demonstrated in a meta-analysis, and are reported to have similar association with incident diabetes (29). A prospective study in Iran also showed WC and WHR to be predictors of incident diabetes (26). Patients with T2DM frequently exhibit an atherogenic lipid profile (high triglyceride and low HDL-C), which increases risk of cardiovascular disease (CVD) compared with normoglycaemia subjects (30). Stern et al. (31) developed two models to predict diabetes incidence. The first was a clinical model that included age, gender, ethnicity, FBG, systolic blood pressure, blood HDL-C level, body mass index and family history of diabetes. The second model included 2-h blood glucose, diastolic blood pressure, blood total and low-density lipoprotein cholesterol, and triglyceride levels. Though not all parameters indicated in the model were used in present study, the models of Stern and colleagues further validate the discourse.

Based on the cost (N 300) of screening for diabetes in not-for-profit hospitals, it would cost N 300,000 to screen 1,000 people to identify <2 individuals with UDM. In view of the socioeconomic cost of DM, consideration needs to be made on the worthiness of spending such an amount to diagnose <2 cases of UDM. Adoption of health economic evaluation in the form of cost-effectiveness informs on priority setting in healthcare (7, 32, 33). A study on cost-effectiveness of screening, which was not aimed to detect diabetes, but those at high risk to develop the disease suggested that such screening in the long term, followed by an adequate programme to promote and support lifestyle changes is cost-effective (34). However, health economic evaluations in terms of assessing cost-effectiveness constitute a vital aspect of deciding whether an intervention is worth implementing. Although people show poor attitude toward voluntary screening, it is necessary to give diabetes screening a priority since studies show that delayed diagnosis may lead to an epidemic (35), and subsequently huge socioeconomic loss (36). Such effect will be profound in SSA, including Nigeria, where the risk of diabetes complications is great and costly (37, 38), and this leads to high health economic burden. To prioritize mass diabetes screening in Nigeria, factors that contribute to the disease such as lack of: knowledge, resources, basic infrastructure and adequate training of health workers as well as poverty need addressing. These factors are possibly responsible for failure to detect DM (39), thus such impediments increase the risk of misdiagnosis and/or delayed diagnosis. This study is also mindful that reliance on traditional, rather than on allopathic medicine is also a major obstacle that is prevalent in SSA (40).

This study observed that FBG remains the best predictor of diabetes, as it showed the least AIC value, which was 4. Given a set of models, the preferred model is the one with the minimum AIC value (41). Such a finding is in line with reports by Cai, Xia (42) who showed that insulin secretion/insulin resistance index was useful as a predictor of development of T2DM. This means that the less insulin secreted to regulate blood glucose level, the more likely it is for blood glucose level to predict diabetes and, vice versa. Indeed, FBG and 2-h plasma glucose are strong predictors of T2DM (43–45), confirming findings of this study.

Blood glucose screening is an important tool to detect diabetes in subjects at risk of developing diabetes or who are asymptomatic. It is debated whether FBG screening is sufficient or oral glucose tolerance test is required to identify asymptomatic diabetes (46). In this study, FBG selectively combined with some parameters to produce models with low AIC (Figure 1). FBG requires an invasive procedure to collect the blood, and it is time consuming as well as expensive especially in LMIC and the test is not informative of long-term glycaemic control. It is suggested that primary prevention entails identification of high-risk subjects in their normoglycaemic state to prevent transition of prediabetes to overt diabetes (47). A concern therefore is that rural dwellers with diabetes are diagnosed late, and by the time diagnosis is made, some of the complications have advanced into irreversible stages (48). In view of the foregoing, it is pertinent to employ model(s) that augment FBG in diabetes screening, since it is not clear if FBG can adequately detect asymptomatic DM.

As identified in this study, a combination of the measured anthropometric and biochemical indices holds promise of detecting DM, especially in asymptomatics since most of the discussed parameters constitute risk factors for diabetes. In rural communities where the infrastructure for blood screening for diabetes lacks and/or where invasive procedures are used, WHR and WC can be used to estimate diabetes risk. The generated models in the study include most of the metabolic syndrome (MetS) parameters as defined by Alberti, Eckel (49). This observation supports the report that people with signs of MetS have increased risk of diabetes (50) and CVD (51). Models involving ODIs had high AIC values, indicating low chance of predicting DM, possibly because the ODIs had not exacerbated to periodontal disease to allow prediction of DM. The interest in this study is a cost-effective screening procedure, with favorable health economic outcomes that can be considered an alternative to FBG, which is expensive and invasive. This will save direct and indirect costs involved in diagnosing diabetes, especially as it concerns community screening. Therefore, anthropometric parameters are perhaps better options to blood lipid profile in predicting diabetes.

## Conclusion

It is established that despite efforts to improve control, DM is not only assuming pandemic proportions globally, and poised to affect developing countries more than developed. Data on health economics of diabetes screening is critical, to provide information on feasible intervention through cost-effective opportunistic screening. Information on the health economic evaluation of managing diabetes and its complications is important for intervention and prevention measures, and it is crucial to consider for policy in early screening and diagnosis of the disease. This report highlights that ODIs have a low probability of predicting DM and thus needs to be used with caution. Anthropometric parameters appear more promising in predicting diabetes, whether in combination with FBG or independently, hence an option to consider for cost-effective diabetes screening. This is imperative in primary healthcare services, especially in rural communities, where facilities for FBG screening may be lacking. Further, this will benefit communities that are considering employing non-invasive screening alternatives for early diagnosis of DM. In the light of health economics, developing further screening programmes for early detection of DM, plus conducting a thorough economic analysis is required for future studies.

## Data availability statement

The raw data supporting the conclusions of this article will be made available by the authors, without undue reservation.

## Ethics statement

The study was carried out in Ndokwa Communities of Nigeria, with the approval (protocol number: 2015/286) of Human Research Ethics Committee (HREC) of Charles Sturt University, Australia. The HREC of Novena University and Ndokwa West Local Ministry of Health Department also approved the research. The patients/participants provided their written informed consent to participate in this study.

## Author contributions

AA, EN, RR, and PB conceptualized the work. EN carried out the analysis of data. AA drafted the manuscript, while EN, RR, and PB reviewed and edited the work. All authors contributed to the article and approved the submitted version.

## Conflict of interest

The authors declare that the research was conducted in the absence of any commercial or financial relationships that could be construed as a potential conflict of interest.

## Publisher's note

All claims expressed in this article are solely those of the authors and do not necessarily represent those of their affiliated organizations, or those of the publisher, the editors and the reviewers. Any product that may be evaluated in this article, or claim that may be made by its manufacturer, is not guaranteed or endorsed by the publisher.
